# Efficacy of 0.05% Chlorhexidine and 0.05% Cetylpyridinium Chloride Mouthwash to Eliminate Living Bacteria on In Situ Collected Biofilms: An In Vitro Study

**DOI:** 10.3390/antibiotics10060730

**Published:** 2021-06-17

**Authors:** Kathrin Becker, Giulia Brunello, Luisa Scotti, Dieter Drescher, Gordon John

**Affiliations:** 1 Department of Orthodontics, University of Düsseldorf, 40225 Düsseldorf, Germany; drescher@med.uni-duesseldorf.de; 2 Department of Oral Surgery, University of Düsseldorf, 40225 Düsseldorf, Germany; giulia.brunello@med.uni-duesseldorf.de (G.B.); luisa.c.scotti@gmail.com (L.S.); gordon.John@med.uni-duesseldorf.de (G.J.); 3 Department of Neurosciences, University of Padua, 35128 Padua, Italy; 4 Dental Practice, 46147 Oberhausen, Germany

**Keywords:** antiseptic, biofilm, cetylpyridnium chloride, chlorhexidine, mouthrinse, mouthwash, peri-implantitis, periodontitis

## Abstract

Chlorhexidine (CHX) mouthwashes are frequently used as an adjunctive measure for the treatment of periodontitis and peri-implantitis, as well as in patients on maintenance therapy. However, their prolonged use is associated with several side effects. This study aimed at evaluating if a mouthwash with a reduced concentration of CHX combined with cetylpyridnium chloride (CPC) was as effective as a conventional CHX mouthwash in the reduction in living cells in oral biofilms attached to hydroxyapatite (HA) and micro-rough titanium (Ti) surfaces. Four healthy volunteers wore a customized acrylic appliance containing HA and Ti discs for in situ plaque accumulation. Biofilms were grown on the discs for 24 or 48 h and then randomly exposed for 60 s to: 0.05% CHX + 0.05% CPC, 0.1% CHX (positive control) or sterile saline (negative control). Viability assay and live-dead staining were performed to quantify bacterial viability and to distinguish live and dead cells, respectively. At both time points, contrary to saline, CHX, both alone and in combination with CPC, exhibited high antibacterial properties and induced a significant reduction in biofilm viability. This study demonstrates the potential of mouthwashes containing a low concentration of CHX combined with CPC as effective antibacterial agents for long-term applications with reduced undesired side effects.

## 1. Introduction

Periodontal and peri-implant diseases are highly prevalent biofilm-associated inflammatory diseases affecting the supportive structure of teeth or dental implants [1,2,3,4,5,6]. Gingivitis and mucositis are reversible lesions. Without treatment, however, they can evolve into the more severe and irreversible periodontitis or peri-implantitis, respectively, characterized by connective tissue inflammation and progressive loss of the supporting bone [7,8].

Many studies demonstrated that plaque accumulation plays a crucial role not only in the onset and progression of both pathologies but also in their recurrence [9,10,11,12]. Self-performed and professionally administered infection control measures are considered essential in the prevention and treatment of periodontal and peri-implant diseases [13,14,15,16,17], as well as in long-term success after disease resolution [18].

Maintenance becomes particularly important when moderately rough implant surfaces are exposed to the oral cavity. They are widely used owing to the favorable bone response [19]; however, they also facilitate microbial adhesion, leading to an increased risk of recurrence [20,21,22,23].

Beside supportive professional maintenance care programs, adequate self-administered daily home care is recommended. This generally includes the use of a toothbrush, toothpaste and interdental tools, as well as mouthwashes, as adjunctive antiseptic measures to disrupt the biofilms [24,25]. Among these, chlorhexidine (CHX) is most commonly used due to its well-documented antimicrobial activity [26]. However, prolonged CHX usage was also reported to be associated with several drawbacks, such as extrinsic tooth staining, taste disturbance/alteration, burning sensation and loss of efficacy overtime [26,27,28,29]. Since these side effects were reported to be dose-dependent [30], low-concentration CHX solutions, combined with other antimicrobials, have been proposed to overcome these drawbacks without losing clinical efficacy [31,32]. Among these adjunctive products, cetylpyridinium chloride (CPC), a cationic surface-active agent belonging to the quaternary ammonium group, is considered to be particularly promising in combination with CHX [33].

Therefore, the goal of the present investigation was to test if a mouthwash with a reduced concentration of CHX (0.05%) and CPC (0.05%) was as suitable as a conventional CHX (0.1%) mouthwash in the reduction in living cells in oral biofilms at hydroxyapatite and micro-rough titanium surfaces.

## 2. Results

This study was performed in four non-smoking, healthy subjects (two females, two males), aged 25–37 years, with good oral hygiene (plaque index <1). In situ plaque collection was performed at 24 and 48 h. The study adhered to the “Strengthening the Reporting of Observational Studies in Epidemiology” (STROBE) guidelines [34].

### 2.1. Viability Assay

**Figure 1:**
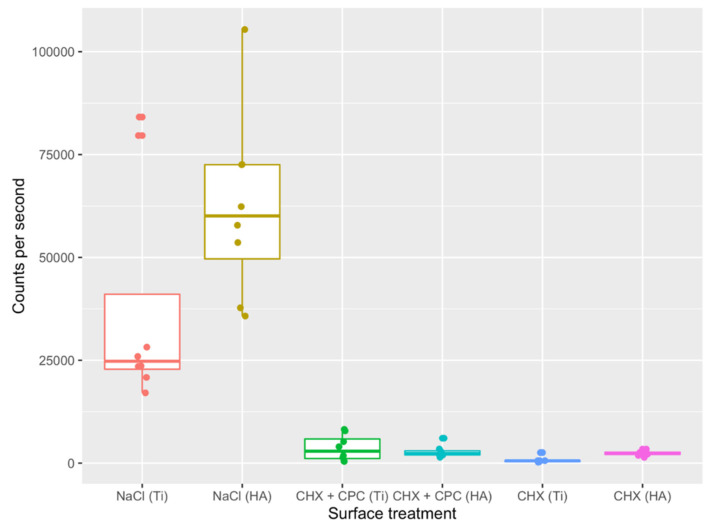
Bacteria viability after 24 h of in situ plaque collection and treatment with NaCl, CHX + CPC or CHX at two types of surfaces (i.e., Ti and HA).

After 24 h, the highest cell counts per second were recorded for the discs rinsed with NaCl (Figure 1), whereas titanium (Ti) and hydroxyapatite (HA) discs treated with 0.05% CHX + 0.05% CPC (CHX + CPC) and 0.1% CHX (CHX) rinses showed very low counts per second (Figure 1). Significant differences were detected between the NaCl (negative control) and the two other groups (CHX + CPC, CHX), whereas significance failed between the latter (test and positive control) for both surfaces (Table 1).

**Figure 2:**
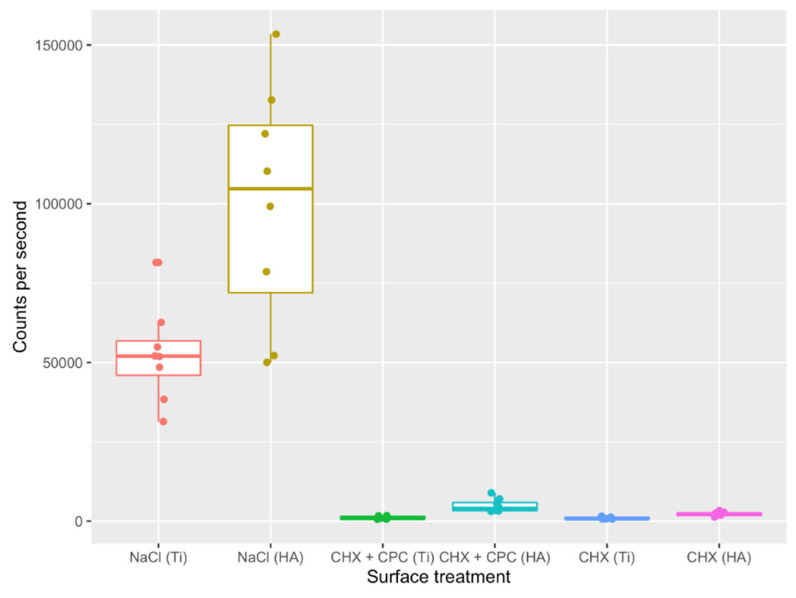
Bacteria viability after 48 h of in situ plaque collection and treatment with NaCl, CHX + CPC or CHX at two types of surfaces (i.e., Ti and HA).

Similarly, after 48 h, the highest cell counts per second were found for the discs rinsed with NaCl (Figure 2), whereas Ti and HA discs treated with CHX + CPC and CHX rinses showed very low counts per second. For Ti surfaces, significant differences were also detected between the NaCl and CHX + CPC and CHX groups, whereas, despite non-overlapping quartile ranges in the boxplot (Figure 2), significance failed between the NaCl (HA) and CHX + CPC (HA) groups (which might be a false negative result owing to the non-parametric test utilized). Additionally, it failed between CHX + CPC and CHX for both investigated surfaces (Table 2).

### 2.2. Live-Dead Staining

**Figure 3:**
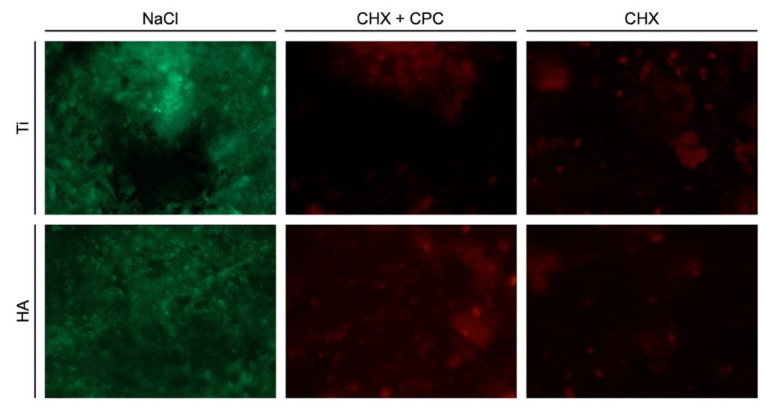
24-h-old biofilm on Ti and HA surfaces after treatment with NaCl, CHX + CPC or CHX.

**Figure 4:**
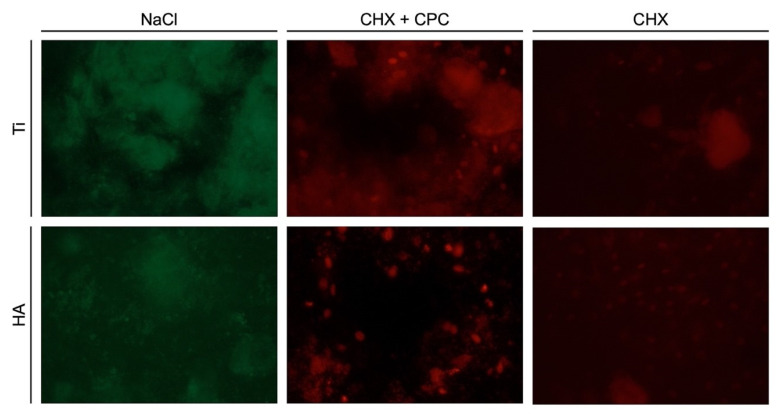
48-h-old biofilm on Ti and HA surfaces after treatment with NaCl, CHX + CPC or CHX.

The live-dead staining procedure allowed distinguishing the living bacteria (labeled in green) from the dead ones (labeled in red). At both 24 and 48 h, no dead bacteria were detected in the NaCl groups regardless of the surface, demonstrating the high viability of the bacterial biofilm (Figure 3 and Figure 4). In contrast, at both time points, almost no living cells could be observed on Ti and HA samples when CHX + CPC and CHX were used. Both solutions showed antimicrobial properties and induced a significant reduction in biofilm viability.

## 3. Discussion

The present study aimed at evaluating the efficacy, in terms of the reduction in vital bacteria, of a mouthwash containing a low concentration of chlorhexidine in combination with cetylpyridinium chloride (CHX + CPC) as compared to the widely used chlorhexidine 0.1% (CHX) mouthwash. In order to mimic the exposure of tooth and implant surfaces to an oral biofilm, discs made of hydroxyapatite (HA) and of a commonly used titanium implant surface (Promote^®^, CAMLOG Biotechnologies AG, Basel, Switzerland) (Ti) were utilized for in situ plaque collection.

At 24 and 48 h, significant differences were recorded between the sterile saline group (NaCl) and the other two groups, i.e., CHX + CPC and CHX, when applied on Ti surfaces. Interestingly, and despite the non-overlapping interquartile ranges, no significant difference was identified between NaCl and CHX + CPC on HA surfaces. However, as no differences were found between the CHX + CPC and CHX groups in all experimental conditions, the present results indicate that both CHX groups demonstrated comparable efficacy.

Cetylpyridinium chloride is a quaternary ammonium compound, included in the group of cationic surface-active agents, and originally, it demonstrated only moderate efficacy [35]. However, when combined with chlorhexidine, a synergistic effect is assumed, increasing the overall antimicrobial activity [36].

Self-administered antiseptic mouthwashes, as an adjunctive measure to mechanical debridement for patients in supportive periodontal care, were frequently reported to be effective in reducing plaque accumulation, in decreasing the proportion of bacteria from the red and orange spectrum and in the reduction in probing depths [37,38]. Few studies investigated the efficacy of the combination of cetylpyridinium chloride and chlorhexidine and demonstrated a reduction in plaque levels and bacterial counts [32], as well as in bleeding on probing (BOP) scores [31]. A double-blind randomized controlled trial (RCT) compared the adjunctive use of 0.05% CPC and 0.05% CHX (with and without alcohol) with 0.2% CHX and found both effective in improving plaque and gingivitis indices [39].

For peri-implant mucositis, the beneficial effect of an antiseptic mouthwash as an adjunctive measure to mechanical debridement remains controversial [40,41]. Two studies (reporting on the same sample of patients) investigated the long-term efficacy, i.e., up to 12 months of follow-up, of 0.03% CHX and 0.05% CPC as an adjunct to professionally and patient-administered mechanical plaque removal in the treatment of peri-implant mucositis. The tested mouthwash resulted in a significant higher reduction in buccal BOP values compared to the placebo mouthwash [42,43]. In both studies, a placebo mouthwash was used as a control in the treatment of peri-implant mucositis, while no comparison was performed with chlorhexidine at higher concentrations.

Therefore, limited evidence exists regarding the efficacy of antiseptic mouthwashes as an adjunctive measure for patients with peri-implant tissue inflammation. The present study demonstrated comparable antimicrobial properties on Ti and Ha surfaces for the combination of 0.05% CPC and 0.05% CHX. In situ plaque collection was selected, as it is considered a useful tool mimicking the normal biofilm development, which is characterized by high complexity and by the presence of numerous bacterial strains [44,45]. By contrast, in vitro cultivation of bacterial biofilms is not likely to mirror the architecture and the composition of the in vivo biofilms. However, specific pathogens can be selected for cultivation that may not be contained in biofilms retrieved from healthy volunteers [44,46]. In the present study, four periodontally healthy volunteers wore acrylic appliances with Ti and HA discs to build up supragingival plaque. Nonetheless, a shortcoming of this approach might consist in the selection of the participants, whose microbiota could differ from that in patients with a history of periodontal or peri-implant disease [47], who represent the target of prolonged use of tested mouthwashes. A larger and more representative pool of participants could be used in future investigations.

Further limitations include the absence of biofilm characterization and of cytocompatibility tests. Regarding the latter, the oral cavity contains different cells, including fibroblasts and epithelial cells. As the mouthwashes are meant to be in contact with the oral mucosa, beside their antimicrobial properties, cell compatibility should also be investigated [48,49].

Finally, corrosion seems to affect dental implants’ biocompatibility, leading to their long-term failure [50]. Although both 0.12% CHX gluconate and 0.5% solutions did not alter the corrosive behavior of sandblasted, acid-etched Ti surfaces in vitro [51], it would be interesting to investigate the effect of the mouthwashes utilized in the present study on commonly used dental implant surfaces.

Furthermore, in relation to the ongoing COVID-19 pandemic, CHX- and CPC-based pre-procedural mouthwashes might also be effective in reducing the risk of SARS-CoV-2 transmission in dental settings, as recently suggested [52,53].

In summary, the present study is in line with previous investigations demonstrating the efficacy of the 0.05% CHX + 0.05% CPC formulation, which permits the use of lower concentrations of CHX while maintaining high antibacterial properties.

## 4. Materials and Methods

### 4.1. Study Population

Four healthy subjects (age ≥ 18 years, non-smokers, good oral hygiene and health, plaque index < 1, no antibiotic therapy within the last 6 months, absence of periodontal diseases as per Papapanou et al. [54]) were included for the collection of the biofilm. All volunteers signed a written consent form before participating in the present study. The study protocol was approved by the Ethics Committee of the University of Düsseldorf (Protocol no. 5797R). The study was conducted following the recognized standards of the Declaration of Helsinki and the European Medicines Agency Guidelines for Good Clinical Practice. The present study was also performed and reported according to the Strengthening the Reporting of Observational Studies in Epidemiology (STROBE) guidelines [34].

### 4.2. In Vivo Biofilm Formation

A customized acrylic appliance for the upper jaw was produced for each subject, containing a range of 27 to 34 discs (2 mm in thickness and 5 mm in diameter) of two different materials, i.e., hydroxyapatite (HA) provided by Dentaid^®^ GmbH (Barcelona, Spain) and titanium (Ti) with moderately rough Promote^®^ surface (CAMLOG Biotechnologies AG, Basel, Switzerland). The subjects were randomly allocated to wear the appliance for either 24 or 48 h to achieve in situ plaque collection. The subjects were permitted to take off the appliance during eating and to perform mechanical tooth brushing without toothpaste or any other chemical adjuncts. The customized appliances were fabricated as described in John et al. [55]. Briefly, the discs were glued in impression to the palatal side of the appliance with a cyanoacrylate glue (Loctide^®^ 496, Henkel AG & Co. KGaA, Düsseldorf, Germany), leaving a 1-mm distance between the palatal mucosa and the disc surface exposed to the oral cavity.

After plaque accumulation for 24 or 48 h, the disks were collected and gently rinsed with sterile water to remove macroscopic food debris and randomly assigned to the following treatment groups: test group 0.05% CPC + 0.05% CHX (PERIO-AID^®^ Active Control, Dentaid^®^ GmbH, Barcelona, Spain) (CPC + CHX), positive control 0.1% CHX (Chlorhexamed^®^ Fluid 0.1%, GlaxoSmithKline Consumer Healthcare GmbH & Co. KG, Bühl, Germany) and negative control (sterile saline). The application time of the mouthwashes was 60 s.

Viability assays were used to quantify bacterial viability. Additionally, live-dead staining was performed for descriptive purposes.

### 4.3. Viability Assay

A total of 96 discs, 8 per group at both time points, were used for the assessment of bacterial viability. Immediately after treatment with the mouthwashes, the discs were transferred to 96-well plates. The bacterial viability was measured using the BacTiter-Glo^®^ luminescent viability assay kit (Promega, Madison, WI, USA), following the instructions of the manufacturer. This test is based on the luciferase-catalyzed reaction of luciferin and adenosine triphosphate (ATP) and, hence, quantifies the ATP present, which indicates the presence of metabolically active cells.

Briefly, 100 μL of BacTiter-Glo^®^ reagent was added to the wells and incubated in darkness at room temperature. The luminescent signal was then recorded using a luminometer (Victor 2030, PerkinElmer, Rodgau, Germany).

### 4.4. Live-Dead Staining

Live-dead staining was performed on 3 samples per group and time point. For fluorescent sample staining, LIVE/DEADTM BacLight^TM^ Bacterial Viability Kit (Thermo Fisher Scientific, Wesel, Germany) was utilized, and photographs were then taken (ColourView III, Olympus Europa GmbH, Hamburg, Germany) using a stereomicroscope (SZ61, Olympus Europa GmbH, Hamburg, Germany).

### 4.5. Statistical Analysis

Statistical evaluation was conducted using the software R [56]. For each time point, surface and mouthwash, boxplots were created for descriptive purposes. The Kruskal-Wallis test and the post hoc multiple comparison Nemenyi test with the Tukey method for *p*-value adjustment were used to assess statistical differences in bacterial viability among the three treatment groups (applied at two surfaces) per time point. The results were found significant at *p* < 0.05.

## 5. Conclusions

In conclusion, within the limitations of the present study, both 0.05% CHX + 0.05% CPC and 0.1% CHX solutions exhibited comparable antibacterial properties when used to rinse hydroxyapatite and titanium surfaces. Due to the reduced concentration of CHX, the combination of CPC and CHX might be beneficial for long-term application.

## Figures and Tables

**Table 1:** A multiple comparison test (Nemenyi post hoc test) was performed to compare the groups after 24 h of in situ plaque collection.

	NaCl (Ti)	NaCl (HA)	CHX + CPC (Ti)	CHX + CPC (HA)	CHX (Ti)
NaCl (HA)	0.98774	-	-	-	-
CHX + CPC (Ti)	0.06271	0.00850 **	-	-	-
CHX + CPC (HA)	0.10432	0.01649 *	0.99996	-	-
CHX (Ti)	0.00014 **	5.9 × 10^6^ ***	0.55798	0.42892	-
CHX (HA)	0.08329	0.01227 *	1.000	1.000	0.48671

The respective *p*-values are provided in the table. Significant values are labeled: * *p* < 0.05, ** *p*< 0.01, *** *p*< 0.001.

**Table 2:** A multiple comparison test (Nemenyi post hoc test) was performed to compare the groups after 48 h of in situ plaque collection.

	NaCl (Ti)	NaCl (HA)	CHX + CPC (Ti)	CHX + CPC (HA)	CHX (Ti)
NaCl (HA)	0.96369	-	-	-	-
CHX + CPC (Ti)	0.00100 **	2.4 × 10^5^ ***	-	-	-
CHX + CPC (HA)	0.76306	0.25638	0.08921	-	-
CHX (Ti)	0.00032 ***	6.1 × 10^6^ ***	0.99981	0.04310 *	-
CHX (HA)	0.11882	0.01087 *	0.69282	0.84726	0.51626

The respective *p*-values are provided in the table. Significant values are labeled: * *p* < 0.05, ** *p*< 0.01, *** *p* < 0.001.

## Data Availability

Data will be provided upon reasonable request.
